# Taxonomic and Ecological Notes on *Termes propinquus* Holmgren, 1914 Known from Sumatra (Blattodea: Termitoidae: Termitidae)

**DOI:** 10.1155/2022/9475722

**Published:** 2022-02-02

**Authors:** Samsul Muarrif, Samadi Samadi, Jauharlina Jauharlina, Dalil Sutekad, Syaukani Syaukani

**Affiliations:** ^1^Graduate School of Mathematics and Applied Sciences, Universitas Syiah Kuala, Banda Aceh 23111, Indonesia; ^2^Department of Animal Husbandry, Faculty of Agriculture, Universitas Syiah Kuala, Banda Aceh 23111, Indonesia; ^3^Department of Plant Protection, Faculty of Agriculture, Universitas Syiah Kuala, Banda Aceh 23111, Indonesia; ^4^Biology Department, Faculty of Mathematics and Natural Sciences, Universitas Syiah Kuala, Banda Aceh 23111, Indonesia

## Abstract

The genus *Termes* Linneus, 1758 consisting of a total of 24 valid named species known from the Old World, is a very heterogeneous group of termites and seems to involve many taxonomic obscurities and confusions. In the island of Sumatra, the sixth-largest island located in the Southeast Asian tropics, four species of *Termes* have been found, namely, *T. comis*, *T. laticornis*, *T. rostratus*, and *T. propinquus*. *Termes propinquus* is also known from Brunei, Indonesia (Kalimantan and Sumatra), Malaysia, and Thailand. However, previous authors have mentioned that *T. propinquus* has been poorly discriminated from the other congeners, especially *T. rostratus*. Therefore, the present study aimed at clarifying the discrimination of *Termes propinquus* from the morphologically similar congeners from Sumatra. A total of 14 nests were collected using a standardized sampling protocol and visual colony searching in Sumatra and its adjacent island. As a result of a careful morphological examination of the soldier caste, *T. propinquus* was discriminated from the three other congeners by a combination of the following characteristics: distinctly long frontal projection, larger head capsule, and 2^nd^ antennal segment distinctly longer than the 3^rd^. The redescription of the soldier caste of *T. propinquus* and a key to *Termes* species known from Sundaland are provided. The nests of *T. propinquus* were attached to the bases of living trees, clinging to stumps or the bases of the dead tree, or were epigeal.

## 1. Introduction

Termites, from the epifamily Termitoidae (or the infraorder Isoptera), are dominant invertebrates in tropical and subtropical soil ecosystems [1–3] and perform indispensable functions as mechanical decomposers and ecological engineers, creating and preparing microhabitats for other organisms, including microbes that contribute to the decomposition process of organic matter [4–6].

Termitoidae is a monophyletic lineage consisting of more than 2 900 validly named species [7, 8], belonging to 281 genera and nine families. Six of these families, namely, Kalotermitidae, Archotermopsidae, Hodotermitidae, Rhinotermitidae, Stylotermitidae, and Termitidae, are known from the Oriental region [9], and three families, namely, Kalotermitidae, Rhinotermitidae, and Termitidae, have been recorded in the Indo-Malayan subregion.

Termitidae constitutes the most species-rich and ecologically diversified family [10]. In Southeast Asia, this family is represented by four subfamilies, namely, Apicotermitinae, Termitinae, Macrotermitinae, and Nasutitermitinae [11, 12]. The genus *Termes* was classified by Holmgren [13] and consists of a total of 24 validly named species, of which most are known from the Ethiopian, Neotropical, and Oriental regions, and a few from the Palearctic region [9]. The soldier caste of the genus has slightly asymmetrical [12] or asymmetrical snapping mandibles and a frontal projection [14]. The classification of the genera of the *Capritermes* complex has been in a confused state because some of the genera, including those involving *Termes*, seem to be composed of heterogeneous lineages [12, 15]. The nesting behavior of *Termes* is also diverse; various shapes of nests are found, and inquilism is also seen [16].

On the island of Sumatra, the sixth-largest island located in the Southeast Asian tropics, four species of *Termes* are known, namely, *T. comis* [17], *T. laticornis* [17], *T. rostratus* [17], and *T. propinquus* [13]. *Termes propinquus* is known from Indonesia (Kalimantan and Sumatra), Malaysia, Brunei, and Thailand, and its type locality is Sumatra (Tandjong Slamat) [9, 13, 15]. However, Holmgren [13] and other authors [18, 19] have mentioned that *T. propinquus* has been poorly differentiated from the other congeners, especially *T. rostratus* [12].

The present article, therefore, aimed to give a redescription of the soldier caste of *T. propinquus* and to clarify the differentiation of *T. propinguus* from the other three Sumatran congeners based on the external morphology of the soldier caste. In addition, information on the nesting behavior of *T. propinquus* is provided.

## 2. Material and Methods

The specimens of *T. propinquus* examined in the present study were collected from various habitats and altitudinal zones of Sumatra between 1998 and 2017 (Table 1). As many as 10–20 individuals of the soldier caste were used for each colony. The syntypes in the collection of the Entomology Department, the Natural History Museum (UK), were examined (Table 2). The focus stacking images of the head, body, pronotum, and antenna of the soldier caste were created using Helicon Focus 6 software based on source images taken as multilayer montages using a Leica M205C stereomicroscope, controlled by Leica Application Suite version 3 software at the Fort Lauderdale Research and Education Center, University of Florida (USA). The images were taken by placing the specimen in a transparent Petri dish filled with ethanol gel (Purell hand sanitizer) in order to keep the specimen at an appropriate angle. A line drawing was also prepared based on the focus stacking images for *T. propinquus*, while *T. rostratus, T. comis,* and *T. laticornis* were redrawn with minor editorial modifications from Tho [12]. General morphological terminology and definitions of measuring characters followed Roonwal and Chhotani [20]. Head capsule length (HL) and maximum head width (HW) were measured by following Roonwal and Chhotani [20], Thapa [19], and Tho [12].

## 3. Result and Discussion

### 3.1. Redescription of *Termes propinquus* Holmgren, 1914 Based on the Soldier Caste

**Figure 1:**
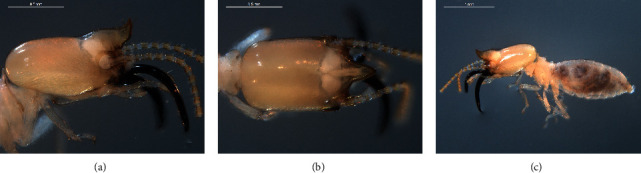
(a) Termes propinquus in lateral view. (b) Termes propinquus in dorsal view. (c) Termes propinquus body in lateral view.

Head capsule brownish yellow; mandibles dark reddish-brown; antenna and pronotum of thorax pale brownish-yellow; and labrum, meso- and metanotum of thorax, abdominal tergites, and legs pale ivory white (Figure 1). Head with several scattered hairs; frontal projection with long hair at the tip and on the dorsal side; postmentum with several hairs along the anterior and anterolateral margins; pronotum with long and short hair along the margins; and abdominal tergites with dense hairs. Head capsule in dorsal view elongate, almost 1.5 times longer than broad, rectangular or subrectangular; lateral margins straight and parallel. Frontal projection very long, in dorsal view cone-shaped; the apex in lateral view upcurved, acutely pointed; anterior margin in lateral view convex (Figures 1(a)–1(c)). Antenna 14-segmented; 2^nd^ longer than 3^rd^; 4^th^ shorter than 3^rd^; 5^th^–14^th^ gradually increased in length toward the apex. Labrum elongate, longer than broad; lateral margin almost straight and parallel; anterior margin sinuate. Mandibles almost symmetrical; left mandible long, slender, gently incurved in the basal half, and acutely incurved at the tip. Postmentum in ventral view slightly swollen posteriorly. Pronotum saddle-shaped; anterior margin without a notch; posterior margin weakly concave at the middle. Legs short; apical tibial spurs 3 : 2 : 2.

### 3.2. Taxonomic Remarks

**Figure 2:**
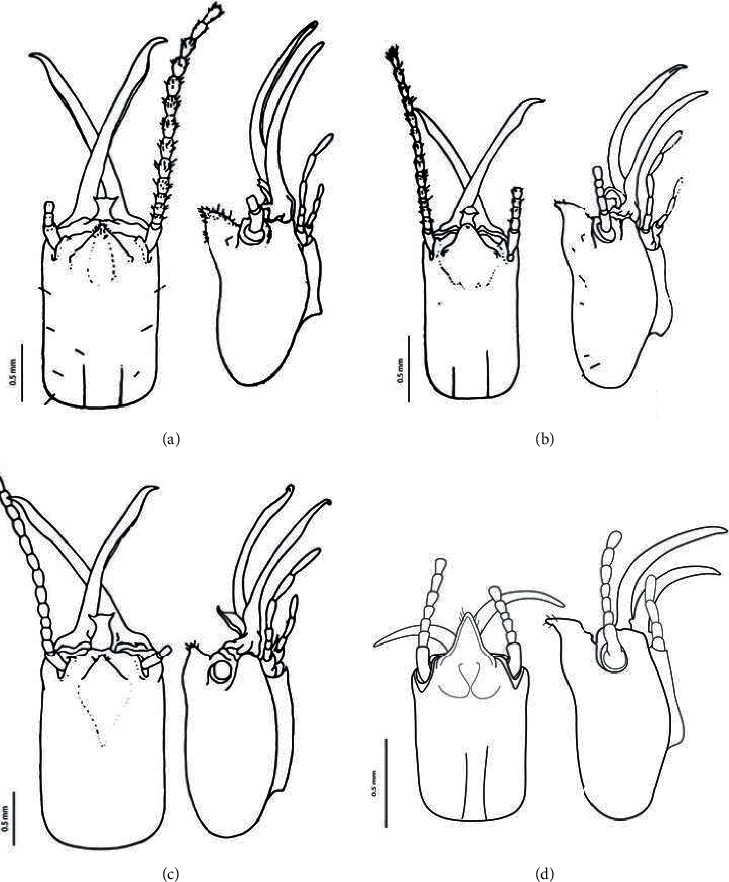
Dorsal and lateral views of head of the soldier caste. (a) Termes comis, (b) T. rostratus, (c) T. laticornis, and (d) T. propinquus. (a‐c) Redrawn with minor editorial modification from Tho (1992), (d) drawn from the specimen (SUAQ-TT-2017-C012).

*Termes propinquus* can be discriminated from the three other congeners from Sumatra based on the external morphology of the soldier caste (Table 2). In the lateral view of the head, the frontal projection is less elongated in *T. laticornis* (Figure 2(c)) than in *T. propinquus* (Figure 2(d)). The head capsule is distinctly larger in *T. laticornis* (HL 1.82 mm, HW 1.13 mm) than in the other congeners from Southeast Asia [12, 19]. In the lateral view, the frontal projection is more elongated in *T. propinquus* (Figure 2(d)) than in *T. rostratus* (Figure 2(b)). The 2^nd^ antennal segment is distinctly longer than the 3^rd^ in *T. propinquus*, but only slightly longer than the 3^rd^ in *T. comis*; the frontal projection in the lateral view is extremely elongate and has sparse hairs in the former (Figure 2(d)), but is moderately elongated and has dense hairs in the latter (Figure 2(a)). A key to the *Termes* species from the Sundaland of Southeast Asia, based on the soldier caste, is provided as below. For HL and HW, the range (*n* = 10) and mean are given.

(i) Lateral view: frontal projection less elongated; HL 1.80–1.85 mm, 1.82 mm; HW 1.00–1.15 mm, 1.13 mm. …………………………….. *T. laticornis* [17]
(ii) Lateral view: frontal projection moderately to extremely elongated …………….. 2
(i) Lateral view: frontal projection extremely elongated; HL 0.95–1.113 mm, 1.12 mm; HW 0.70–0.73 mm, 0.72. …………………..…….. *T. propinquus* [13]
(ii) Lateral view: frontal projection moderately elongated ………………………..…….. 3
(i) Dorsal view: frontal projection with dense hairs (especially on the anterior face); HL 1.40–1.45 mm, 1.44 mm; HW 0.90–1.10 mm, 0.94 mm. ………….… *T. comis* [17]
(ii) Dorsal view: frontal projection with sparse hairs; HL 1.00–1.20 mm, 1.13 mm; HW 0.60–0.72 mm, 0.68 mm. ………………………………………. *T. rostratus* [17]

### 3.3. Nesting Behavior

**Figure 3:**
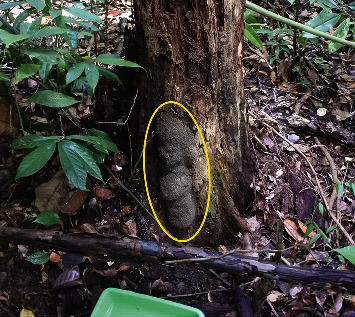
A nest of Termes propinquus attached to a stump, in a diptercarp forest of Suaq Balimbing, North Sumatra (Suaq Balimbing Field Station; colony (SUAQ-TT-2017-C012).

A total of 14 nests of *T. propinquus* [13] were found in this study (Table 1 for colonies and nests). Four nests (28%) were attached to the bases of living trees, and seven nests (50%) were attached to stumps or the bases of dead trees (Figure 3). The advantages of this type of nest might be (1) to increase the physical robustness of the nests (resistance to disturbances by ground-dwelling mammals or heavy rain), (2) to economize the total amount of building material for building nests, (3) to reduce dehydration by reducing the surface area of the nests, and (4) to gather food and building materials, such as rotten wood and humus, which accumulate around the tree base and stumps.

**Figure 4:**
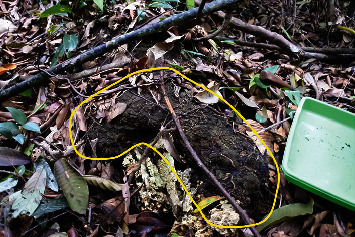
A rebuilt nest of Termes propinquus after being destroyed probably by a mammal, in a diptercarp forest of Suaq Balimbing, North Sumatra (Suaq Balimbing Field Station; colony (SUAQ-TT-2017-C022).

Three nests (22%) were epigeal (standing on the ground); however, epigeal nests might be destroyed by ground-dwelling mammals (Figure 4). Mammals are probably ranked as the second most important predators for termites; the first position is likely occupied by ants [21–23]. We also found as many as four of the 14 epigeal nests showed signs of having been attacked by ants, such as *Camponotus gigas* (two nests), *Pheidole* sp. (one nest), and *Oecophylla smaragdina* (one nest). Ants prey on termites and also often destroy their nests. The irregular-shaped nests clinging to the forest floor (Figure 4) are likely to be rebuilt from destroyed epigeal nests. Therefore, multiple physical and biotic factors on the forest floor might influence the diversity of the nest architecture of *T. propinquus*.

## 4. Conclusion

As many as four species of the genus *Termes* have so far been recorded from Sumatra, including *Termes propinquus* that can be separated from other congeners based on the condition of frontal projection, the head capsule structure, and the antennal segmentation in the soldier caste. Measurements of the head capsule are useful for species identification to the Sumatran genus. *Termes propinquus* prefers to construct their nests attached to bases of living or dead trees, epigeal, and stumps. Both ants and mammals were the most important predators of *T*. *propinquus* on Sumatra.

## Figures and Tables

**Table 1:** Examined colonies of *Termes propinquus* collected from Sumatra and adjacent islands.

Colony ID	Sampling site			Forest type	Nest type
	Abb.	Coordinates	Altitude (m)		
KMR-SYK-1999-C015	NS-KM	03° 47′ 50″ N 97° 33′ 02″ E	1,100	Primary submontane	Attached to the bases of living trees
KMR-SYK-1999-C008	NS-KM	03° 47′ 50″ N 97° 33′ 02″ E	1,100	Primary submontane	Clung to the stumps or the bases of dead tree
KMR-SYK-2000-C106	NS-KM	03° 47′ 50″ N 97° 33′ 02″ E	1,100	Primary submontane	Clung to the stumps or the bases of dead tree
KMR-SYK-2014-C018	NS-KM	03° 47′ 50″ N 97° 33′ 02″ E	1,100	Primary submontane	Clung to stumps or the bases of dead tree
SE-FC-2016-C027	NS-SE	05° 26′ 4″ N 95° 41′ 47″ E	500	Secondary low land dipterocarp	Clung to the stumps or the bases of dead tree
KMR- SYK-2014-C022	NS-KM	03° 47′ 50″ N 97° 33′ 02″ E	1,100	Primary submontane	Epigeal
SUAQ- SYK-1999-C011	NS-SB	03° 02′ 51″ N 97° 25′ 01″ E	100	Primary low land dipterocarp	Epigeal
SUAQ-TT-2017-C012	NS-SB	03° 02′ 51″ N 97° 25′ 01″ E	100	Primary low land dipterocarp	Clung to the stumps or the bases of dead tree
SUAQ-TT-2017-C022	NS-SB	03° 02′ 51″ N 97° 25′ 01″ E	100	Primary low land dipterocarp	Epigeal
BL-SYK-2002-C061	NS-BL	03° 32′ 18″ N 98° 08′ 50″ E	350	Primary low land dipterocarp	Attached to the bases of living trees
BL SYK--2014-C034	NS-BL	03° 32′ 18″ N 98° 08′ 50″ E	350	Primary low land dipterocarp	Attached to the bases of living trees
KSNP SYK--2006-C-27	WS-KS	00° 41′ 32″ S 100° 26′ 36″ E	1,200	Primary submontane	Clung to the stumps or the bases of dead tree
SNP- SYK-2000-C014	MT-SB	01° 29′ 22″ S 98° 58′ 09″ E	50	Primary low land dipterocarp	Attached to the bases of living trees
SNP-SYK-2007-C098	MT-SB	01° 29′ 22″ S 98° 58′ 09″ E	50	Primary low land dipterocarp	Clung to the stumps or the bases of dead tree
SI-SYK-2007-006	MT-PS	02° 11′ 57″ S 99° 40′ 02″ E	50	Secondary low land dipterocarp	Clung to the stumps or the bases of dead tree

Abbreviations (Abb.) of sampling sites: NS-KM, Kemiri, North Sumatra; NS-SE, Seulawah Ecosystem, North Sumatra; NS-BL, Bukit Lawang, North Sumatra; NS-SB, Suaq Balimbing, North Sumatra; WS-KS, Kerinci Seblat, West Sumatra; MT-SB, Siberut, Mentawai; MT-PS, Pulau Sipora, Mentawai.

**Table 2:** Morphological comparison among four *Termes* species known from Sumatra and adjacent islands.

Species	Measurement range (*n* = 10) and mean		Frontal projection	
	HL	HW	Lateral view	Dorsal view
*Termes comis* [17] Syntype no.305	1.40–1.45, 1.44	0.90–1.10, 0.94	Moderately prominent, with dense hairs especial on the anterior face	Medium size, cone-shaped
*T. laticornis* [17] Syntype no. 231	1.80–1.85, 1.82	1.00–1.15, 1.13	Weakly prominent, with sparse hairs	Small size, cone-shaped
*T. rostratus* [17] Syntype no. 477	1.00–1.20, 1.13	0.60–0.72, 0.68	Moderately prominent, with sparse hairs	Small size, dome-shaped
*T. propinquus* [13]	0.95–1.13, 1.12	0.70–0.73, 0.72	Strongly prominent, with sparse hairs	Extremely large size, cone-shaped

HL, head capsule length; HW, maximum head width.

## Data Availability

The data used to support the findings of this study are available from the corresponding author upon request.
